# Community needs assessment of key populations-at-risk of HIV/AIDS in Nigeria's capital territory

**DOI:** 10.1186/1471-2334-14-S2-P29

**Published:** 2014-05-23

**Authors:** Godwin Asuquo, David Owolabi, Ali Onoja, Uche Okoro, Floria Durueke

**Affiliations:** 1 Africa Center for Health Care Leadership Development, Abuja, Nigeria

## Introduction (aim)

Nigeria currently has one of the highest HIV and AIDS epidemic burden worldwide. Prevalence among key populations is currently, 34.1% (brothel-based sex workers), 21.95 (non-brothel-based), 37.6% (MSMs) and 9.3% for IDUs respectively. A huge gaps exists in the national response to key populations as a result of socio-cultural barriers and unfavorable political and legislative environment. This has become accentuated by the recently enacted anti-gay law. The Federal Capital Administration in collaboration with UNDP and UNFPA in 2013 commissioned a study on key populations in the Capital territory to identify services available to them, the gaps and opportunities for scaling up municipal responses for MSMs, FSWs and IDUs.

## Materials and methods

A cross sectional study relying on the needs assessment methodology which included a community social survey, community mapping and focus group discussions. A total of 120 (70 sex workers, 30 MSMs and 20 IDUs) participated in the study. In-depth interviews were conducted for policy makers and technical leads from government agencies, NGOs and development partners. Institutional analysis of relevant NGOs preceded a desk review of the response to key populations in Nigeria and a validation workshop was conducted to validate the findings.

## Results

About 17,117 FSWs were mapped in 1004 hotspots, while, 692 MSMs were located in 692 hot spots and 135 IDUs in 16 hotspots in the FCT. About 689 non-governmental organizations were providing services with the majority (40%) providing HCT services, 34 %, condom programming, 20 % peer education and 16%, youth friendly services. None of the NGOs was providing STI services while only 10% provided lubricants. HIV/AIDS risk perception and current knowledge of prevention of sexual transmission was generally low (32%). Social stigma, poor coordination and inadequate government support were identified as major barriers to scaling up services.

## Conclusion

The findings support the need for the development of a municipal action plan for key populations in the FCT based on the identified priorities and the review of the national HIV/AIDS strategy to be more responsive to the needs of key populations.

